# Effects of Glutamine Supplementation and Early Cold Conditioning on Cold Stress Adaptability in Broilers

**DOI:** 10.3390/ani15101386

**Published:** 2025-05-11

**Authors:** Hanan Al-Khalaifah, Samar A. Tolba, Afaf Al-Nasser, Ahmed Gouda

**Affiliations:** 1 Environment and Life Sciences Research Center, Kuwait Institute for Scientific Research (KISR), Kuwait City 13109, Kuwait; hkhalifa@kisr.edu.kw (H.A.-K.);; 2 Department of Nutrition and Clinical Nutrition, Faculty of Veterinary Medicine, Zagazig University, Zagazig P.O. Box 44511, Egypt; 3 Department of Animal Production, National Research Center, El Buhouth St., Dokki, Giza P.O. Box 12622, Egypt

**Keywords:** antioxidant activity, broilers, cold conditioning, glutamine, immunity

## Abstract

Cold stress disrupts broiler homeostasis, and a single intervention may be insufficient for protection. This study examined the effects of early cold conditioning (25 ± 1 °C for 3 h at 5 days) and glutamine (Gln) supplementation on broiler thermotolerance during a 16 ± 1 °C cold challenge at 35 days. A 2 × 3 factorial design assigned 360 Cobb-500 broilers to six treatments (six replicates/treatment, ten birds/replicate) with three Gln levels (0%, 0.3%, and 0.5%) and two temperature conditions: standard temperature and a 7 °C reduction at 5 days old. Supplementing with 0.3% and 0.5% Gln and cold conditioning improved growth performance (*p* < 0.05), except for feed intake during the grower-finisher phase and overall growth with cold conditioning alone. Adding 0.3% and 0.5% Gln enhanced hemoglobin, total protein, albumin, triiodothyronine (T_3_), thyroxine (T_4_), antioxidant capacity, catalase (CAT), superoxide dismutase (SOD), heat shock protein 70 (HSP70), interleukin 2 (IL2), IL10, IL4, interferon-γ (INF-γ), and troponin-T levels (*p* < 0.05). Cold conditioning influenced packed cell volume, T_3_, T_4_, CAT, HSP70, IL10, INF-γ, and troponin-T levels (*p* < 0.05). Diet-temperature interaction influenced growth, antioxidant, and immune responses, but not hematological or biochemical indicators. Overall, dietary Gln at 0.3% and 0.5% and early cold conditioning may serve as complementary strategies to mitigate cold stress in broilers.

## Simple Summary

As climate change leads to increasing temperature fluctuations, the poultry industry must develop effective strategies to protect birds and ensure sustainable production. Newly hatched chickens in cold climates often endure cold stress, leading to physiological and metabolic disruptions that hinder growth and reduce production efficiency. No single anti-stress agent can effectively counteract the wide-ranging effects of cold stress on poultry. This study evaluated the combined effects of early cold conditioning and dietary glutamine (Gln) supplementation on broilers exposed to cold stress later in life. Dietary supplementation with 0.3% and 0.5% Gln and cold conditioning improved broiler growth performance throughout the experiment, except for feed intake during the grower-finisher phase and overall growth under cold conditioning alone. Adding 0.3% and 0.5% Gln increased hemoglobin, total protein, albumin, triiodothyronine (T_3_), thyroxine (T_4_), antioxidant capacity, catalase (CAT), superoxide dismutase (SOD), heat shock protein 70 (HSP70), interleukin 2 (IL2), IL10, IL4, interferon-γ (INF-γ), and troponin-T levels; however, cold conditioning affected only blood packed cell volume, T_3_, T_4_, CAT, HSP70, IL10, INF-γ, and troponin-T. These findings suggest that dietary Gln supplementation, along with early cold conditioning, may enhance broiler resilience to cold stress impacts and improve performance, physiological, antioxidant, and immunological responses.

## 1. Introduction

In the context of global warming, the environment becomes more complicated and unpredictable, and animal disturbances grow more common. Currently, low environmental temperatures are one of the most common stressors in cold regions and throughout the winter season, therefore threatening the animals’ health [1,2]. Earlier literature indicated that physiological stress resulting from detrimental climatic circumstances might significantly impact the reproductive and productive performance, health status, and immunological responses of poultry chickens [3,4]. Cold stress has been shown to retard the development and growth of chickens, necessitating increased feed intake to sustain body temperature, which leads to a diminished feed conversion rate and resource wastage, along with suboptimal weight uniformity and carcass quality. Furthermore, mortality and morbidity rates are aggravated [5]. Additionally, cold stress may induce disorders such as ascites, edema, epithelial injury in the intestinal mucosa, and heightened vulnerability to *Escherichia coli* infections during the first few days post-hatch [6,7,8].

“Acclimation” is a term that indicates the physiological modifications that an organism undergoes to increase its resilience to the stressful aspects of its environment [9]. Chickens can be endowed with enhanced thermal stress tolerance during the prenatal and early postnatal periods through an adaptation mechanism known as genomic imprinting, which serves to pre-adapt the organism for the anticipated environmental conditions it will encounter later in life [10,11]. This can also be accomplished during the early postnatal period through the application of thermal conditioning [12]. The acquisition of thermotolerance and the enhancement of performance represent two opposing processes; consequently, the acclimation process is characterized by a temporary decline in performance [13]. Nonetheless, there is a certain period during which thermotolerance may be enhanced through thermal conditioning without compromising performance [14]. During this time frame, the thermoregulatory feedback mechanism is not fully developed, and thermal conditioning can create an enduring “memory” that enhances the capacity to manage thermal stresses in later stages of life [12,14]. Cold conditioning during the early postnatal period has elicited effective responses to cold in poultry. For instance, intermittent cold exposures during the first three weeks of bantam chicks’ lives resulted in a significantly higher growth rate than the controls from the second week onwards [15]. Moreover, after adjusting to a cold environment of 15 °C for many hours, broiler chicks of 3 to 4 days (d) old become more resistant to low-temperature environments [16]. In other mammalian species, rats that had acclimated to chronic moderate restraint stress for 2 to 10 d showed considerably less degree of stomach mucosal damage than their control relatives when they were concurrently subjected to cold stress [17]. In essence, cold acclimation is thought to increase cold tolerance and hasten body temperature recovery in cold environments [18].

Indeed, the stress response is a non-specific systemic adaptive response, and its physiological consequences are various. Thus, it is challenging to maintain body homeostasis through a singular tactic. Consequently, a viable approach is to improve the diet and investigate the protective effects of nutritional additives on cold-stressed chicks in conjunction with the acclimation to cold stress during the first few days post-hatch. We elected glutamine (Gln) to enhance the nutritional and metabolic energy content of the diet in order to mitigate the adverse effects of cold stress. Gln is the most prevalent free amino acid (AA) in the body, and apart from its significance in AA transamination and as a protein constituent, Gln has a variety of nonnutritive functions and has been asserted to be essential in certain clinical or stress-related situations [19,20]. For small intestine enterocytes, macrophages, lymphocytes, and fibroblasts, for instance, Gln is the main metabolic fuel and is regarded as an essential AA in several species under inflammatory circumstances, including infection and damage. Moreover, it is among the most effective substrates for gluconeogenesis, which also supports intestinal tract physiology and function [21,22]. Despite the fact that Gln is a superb supply of both carbon and nitrogen for the synthesis of AA in chicks through amination and amidation, the national research council and the nutritional specification standards for poultry strains have not recommended dietary requirements of poultry for Gln. Nevertheless, prior research had demonstrated that poultry require Gln in their diets to achieve optimal health and growth [23,24]. More crucially, given the low activity of arginase and proline oxidase in tissues of chickens for Gln synthesis from arginine and proline [25,26], the dietary requirements for Gln should be taken into account and are probably greater in birds than in mammals [27]. Through extensive research that has demonstrated that dietary supplementation with Gln can improve the health and development performance of poultry, additional lines of evidence have been provided to support the hypothesis of insufficient Gln synthesis in birds. At first, the feed intake (FI), body weight gain (BWG), and feed conversion ratio (FCR) of 1-day-old broiler chickens were improved by dietary supplementation with 0.4, 0.6, or 0.8% Gln over a 35-day period, without any adverse effects on the carcass percentage [28]. Furthermore, in addition to reducing post-hatching mortality in 1- to 42-day-old broilers under heated and humid conditions, dietary supplementation with 0.5–1% Gln increased the height of the small-intestinal villus, BWG, and feed efficiency [29]. It is significant that Fathi et al. [30] found that the rate of mortality and hepatic injury was reduced in broiler chickens with cold-induced ascites that were 1- to 42-day-old when they were dietary supplemented with 100 ppm Gln. Thus, in order to optimize poultry growth and feed efficiency, it is essential to provide sufficient dietary Gln, particularly under stress conditions. Hence, the present study aimed to evaluate the impact of early-life exposure of broilers to low ambient temperatures, supplemented with dietary Gln, as strategies for coping with cold stress. We have evaluated the effects on broilers’ growth performance, metabolic status, antioxidant capacity, and immune status.

## 2. Materials and Methods

### 2.1. Birds, Diets, and Management

The present experiment was conducted by the Department of Animal Production, the National Research Center, and the Department of Nutrition and Clinical Nutrition, Faculty of Veterinary Medicine, Zagazig University, during the winter season. The Animal Protocol was approved by the Institutional Animal Care and Use Committee of Zagazig University (ZU-IACUC). A total of 360 unsexed Cobb-500 broiler chicks, acquired from a commercial hatchery (Dakahlia Poultry, Mansoura, Egypt), were randomly allocated into six treatment groups (6 replicates per group, 10 birds per replicate) in a 2 × 3 factorial design with an initial average BW of 44.37 ± 0.99 gm. This included three dietary levels of Gln (0%, 0.3%, and 0.5%) and two temperature conditions: standard temperature, set at optimal levels according to the broiler management guide [31], and a cold temperature, which involved a decrease of 7 °C for three hours on day five (25 ± 1 °C), after which the temperature was restored to standard levels. The birds under standard temperature conditions were kept in a normal thermal environment from days 1 to 35, i.e., 32 °C during the first week of age, 30 °C on day 7, and 27 °C on day 14, then the temperature was reduced gradually by 1 °C every 2 days till it dropped to 23 °C and was maintained until the end of the study [31]. The chicks were raised in a hierarchically designed cage system partitioned by wire mesh barriers with dimensions of 1.0 m × 0.50 m × 0.40 m under uniform sanitary and managerial conditions over the experimental period. On the first day of placement, a 24 h light period was administered, followed by a lighting regimen of 23 h of light and 1 h of darkness for the subsequent three days, transitioning to an 18:6 h light-to-dark period until the end of the experiment. For five hours on day 35, all chicks underwent acute cold stress at a 6–8 °C drop in the rearing temperature (16 ± 1 °C) to explore the resistance of the broilers to cold stress. Birds were slaughtered within 6 h after the end of the cold stress.

L-Glu was obtained from Amresco (Solon, OH, USA), with a purity of over 99.0%. The basal diet was corn-soybean meal, and the birds were fed the starter diet until they were 21 days of age, followed by a grower-finisher diet from days 22 to 35. The Gln used was a white crystalline powder. Small amounts of the basal diet were mixed first with quantities of the supplemental glutamine as small batches, followed by a larger quantity of the diets until the whole diet was homogenously mixed. All experimental diets were designed in accordance with the broiler nutrition specifications [31,32] (Table 1). For the whole study, fresh water and feed in a mash form were given ad libitum.

### 2.2. Growth Performance

Upon arrival, the birds were weighed individually to determine their average initial body weight (BW). The BW was subsequently determined at 21 and 35 days, and the BWG was calculated as W2 − W1. The FI for each replicate was calculated by subtracting the weight of leftover feed from the initial feed weight and then dividing this value by the average BWG to derive the FCR [33].

### 2.3. Sample Collection and Biochemical Analysis

At the end of the experiment (35 days), 6 birds from each of the treatment groups were randomly selected within 6 h after the end of the last cold stress for sample collection. For hematological analysis, a 1 mL blood sample was obtained from each bird using heparinized needles by wing vein puncture, thereafter transferred to sample tubes, and immediately kept on ice in a chilled container. Hemoglobin (Hb) and packed cell volume (PCV) were evaluated within two hours of sample collection using the methodologies of Jain [34]. For the biochemical examination, additional blood samples were collected from the same birds into vacutainer tubes absent of anticoagulant (BD Vacutainer, Becton Dickinson, Franklin Lakes, NJ, USA), which were allowed to coagulate at ambient temperature. The samples were subsequently centrifuged for 13 min at 3000× *g*. The supernatants (serum samples) were obtained in Eppendorf tubes and kept at −20 °C until subsequent analysis. Subsequent to blood collection, the same birds were slaughtered by cervical dislocation in accordance with the institutional committee’s recommendations, eviscerated, and a piece of the liver tissue was taken out, vacuum packed, and stored at −20 °C for heat shock protein 70 (HSP70) testing.

Serum total protein (TP) content was determined using the final point biuret method, whereby proteins interact with copper sulfate in an alkaline sodium hydroxide medium to produce a violet-colored biuret complex. The color intensity, assessed spectrophotometrically at 546 nm, is directly proportional to the protein content [35,36]. Serum albumin was measured using a dye-binding approach predicated on the capacity of albumin to establish a stable complex with bromocresol green (BCG) dye. The BCG-albumin complex demonstrates an absorbance change relative to the unbound dye, with absorbance measured at 620 nm [37,38]. Globulin concentrations were obtained by subtracting albumin from TP. The technique employed by Sánchez-Carbayo et al. [39] was utilized to evaluate blood triiodothyronine (T3) and thyroxin (T4) levels by commercially available kits (Byk-Sangtec Diagnostica, Dietzenbach, Germany; Immulite 2000, DPC, Los Angeles, CA, USA) per the manufacturers’ instructions.

### 2.4. Antioxidant Status Assessments

The serum total antioxidant capacity (TAC) was evaluated colorimetrically with commercially available kits (Rel Assay, Şehitkamil/Gaziantep, Turkey) in accordance with the methodology of Janaszewska and Bartosz [40]. Antioxidants present in the serum convert the dark blue-green 2,2′-azinobis (3-ethylbenzothiazoline-6-sulfonic acid) diammonium salt (ABTS) radical into a colorless reduced type of ABTS. The variation in absorbance at 660 nm correlates with the sample’s TAC. The activities of superoxide dismutase (SOD) and catalase (CAT) enzymes were evaluated in the serum using the methodologies provided by Kraljević et al. [41]. The measurement of SOD was premised on the formation of superoxide radicals resulting from the interaction between xanthine and xanthine oxidase, which then produced formazan dye via a reaction with nitro blue tetrazolium. The reduction rates of superoxide anion exhibit a linear correlation with the activity levels of xanthine oxidase, which is inhibited by the SOD and measured at 560 nm. The breakdown of hydrogen peroxide (H_2_O_2_) was monitored at 240 nm in the presence of CAT. The activity of CAT was quantified as the quantity of enzyme required to decompose 1 mmol of H_2_O_2_ per minute at pH 7.8 and 25 °C.

For HSP70 assessment, liver tissue samples (*n* = 6/treatment ~1 g each) were washed three times to eliminate remaining blood and thereafter homogenized in 4 mL of protein buffer using a Polytron Homogenizer (Heat System UltraSonics, New York, NY, USA) for quantification of proteins. The homogenates were subjected to centrifugation at 3000× *g* for 15 min at 4 °C, after which the supernatants were collected for TP analysis. The HSP70 evaluation was conducted in duplicate with commercial enzyme-linked immunoassay kits (Quantikine, R&D Systems, Minneapolis, MN, USA) in accordance with the manufacturer’s instructions. The standard HSP70 level was calculated by dividing the optical density of each sample by its original TP concentrations.

### 2.5. Immune Status Assessments

ELISA kits (Wuhan Fine Biotech Co., Ltd., East Lake High-tech Development District, Wuhan, Hubei Province, China) that are specific for chickens were employed to evaluate the serum levels of proinflammatory cytokines, interleukin 2 (IL2) and interferon-γ (IFN-γ), and anti-inflammatory cytokines, interleukin 10 (IL10) and interleukin 4 (IL4). An ELISA plate reader (benchmark plus microplate spectrophotometer system with incubator, Bio-Rad Laboratories, Hercules, CA, USA) measured optical densities of kit standards and test samples at 450 nm. All procedures were carried out in line with the manufacturer’s guidelines. Following the manufacturer’s recommendations, serum concentrations of Troponin-T have been measured by electrochemiluminescence immunoassay technique (Elecsys 2010, Roche Diagnostics, Mannheim, Germany) [42].

### 2.6. Statistical Analysis

All data derived from the 2 × 3 factorial experimental design (comprising two temperatures and three dietary Gln levels) were analyzed using SPSS software (Version 28.0 for macOS 10.15, Chicago, IL, USA). The main effects of temperature, Gln supplementation, and their interactions were analyzed using the General Linear Model procedure in SPSS. The pen was considered the experimental unit for growth performance measurements, while individual blood draws and health status constituted the experimental unit for blood physiology, antioxidant, and immunological parameters. Significant treatment effects among dietary groups were identified using Tukey’s honestly significant difference test. The differences between the standard temperature and cold-conditioned treatments were examined using Student’s *t*-test. The significance level was set at *p* < 0.05, and results were presented as mean ± standard deviation.

## 3. Results

### 3.1. Growth Performance

Table 2 presents the growth results for the starter, grower-finisher, and overall periods. During the starter period, all growth performance metrics were influenced by the diet and temperature factors. Dietary Gln and cold conditioning led to enhancements in BW, BWG, FI, and FCR (*p* < 0.05) than those in the control group. The interaction effects results indicated that the highest BW was recorded in the cold-conditioned treatment supplemented with 0.3% and 0.5% dietary Gln (*p* = 0.014). Throughout the grower-finisher period and overall performance, BW, BWG, FCR, and FI were improved by the inclusion of 0.3% and 0.5% dietary Gln (Gln) (*p* < 0.05). BW and BWG were enhanced due to cold conditioning throughout the growing period; furthermore, BW, BWG, and FCR all exhibited improvement as a result of cold conditioning during the overall period (*p* < 0.05). Regarding the interaction effect, all treatment groups showed enhanced BW, BWG, and FCR compared to the control diet at standard temperature (*p* < 0.05).

### 3.2. Blood Hematological and Biochemical Parameters

Table 3 presents data regarding hematological and biochemical blood parameters. Blood Hb level and serum TP, albumin, T_3_, and T_4_ were increased in response to 0.3% and 0.5% dietary Gln (*p* < 0.001). The temperature influence on PCV, T_3_, and T_4_ levels was noted, with increases found with cold conditioning (*p* < 0.05). No interaction effects were seen between the diet and temperature factors on any evaluated parameters (*p* > 0.05).

### 3.3. Oxidative Stress Response

Table 4 shows the data related to the antioxidant status. The serum levels of TAC, CAT, and SOD, as well as liver HSP70, were increased in response to 0.3% and 0.5% dietary Gln (*p* < 0.05). Only the levels of HSP70, CAT, and SOD were elevated in response to cold conditioning (*p* < 0.05). Regarding interaction effects, all treatment groups exhibited enhanced TAC and CAT levels compared to the control diet at standard temperature (*p* < 0.05); however, 0.3% and 0.5% Gln supplementation at cold or normal temperature elevated HSP70 levels (*p* = 0.002).

### 3.4. Immune Response

Table 5 shows the data related to immune status. The serum levels of IL2, IL4, IL10, and INF-γ, as well as troponin-T levels, were increased in response to 0.3% and 0.5% dietary Gln (*p* < 0.001). Only IL10, IFN-γ, and troponin-T levels were elevated in response to the cold conditioning (*p* < 0.05). The interaction effects results indicated that cold conditioning combined with dietary Gln supplementation at 0.3% and 0.5% enhances IL4, IL10, INF-γ, and troponin-T levels relative to other treatment groups (*p* < 0.05).

## 4. Discussion

Sudden extreme cold stress can be detrimental to chickens, since it has been proven to elevate basal metabolic rate and energy metabolism, which are critical and energy-intensive for homeotherms [43]. Nevertheless, despite being endothermic, chickens may employ adaptive mechanisms to mitigate cold stress and restore thermal homeostasis [44]. Our present study revealed that cold conditioning of broilers at an earlier age in conjunction with sufficient dietary inclusion of Gln led to enhanced growth performance. Earlier research reported that cold stress adversely impacted chickens’ growth performance with an elevated FCR, suggesting the redistribution of nutrients from growth toward thermoregulatory responses [5,45]. To our knowledge, there is a paucity of prior research on the mutually beneficial effects of cold conditioning and supplemental dietary Gln in broilers as a strategy to mitigate cold stress. Nonetheless, previous studies indicated that the growth performance of broilers subjected to low ambient temperatures was enhanced through Gln supplementation [46]. In our current investigation, dietary Gln supplementation seemed to ameliorate the disrupted energy balance of broilers exposed to cold, facilitating thermoregulatory adaptations established by early cold conditioning, thereby defending the broilers from subsequent stressors later in life. The premise is corroborated by the results of the assessed hematological and biochemical blood indices, as we detected an increase in the majority of the evaluated indicators. These data suggest an improvement in broilers’ health due to the combined effects of cold conditioning and supplemental dietary Gln, which has demonstrated improved growth performance.

The current study attempted to gain a better understanding of the physiological and metabolic processes associated with the enhancement of performance and overall health in birds in response to the combined effects of early cold conditioning and supplemental dietary Gln. The emergence of thermoregulation in bird species is a complicated matter involving both neurological and hormonal processes. In this setting, blood biochemical indicators are often used to assess these responses and evaluate the heat tolerance of broiler chicks. Our present investigation demonstrated improvements in Hb, blood biochemical markers (albumin, TP), and thyroid hormones (T_3_ and T_4_). Cold exposure has been demonstrated to stimulate the hypothalamus, resulting in elevated synthesis and release of thyroid-releasing hormone. This, in turn, stimulates the pituitary gland to produce thyroid-stimulating hormone, which binds to receptors in thyroid follicular cells, thereby increasing the synthesis and secretion of thyroid hormones [47,48]. Thyroid hormone levels are intricately associated with metabolic rate. They increase oxygen consumption and heat production; moreover, they promote the metabolism of proteins, carbohydrates, and lipids, consequently promoting the growth and development of the animal [47]. Prior research indicated that feeding Gln resulted in elevated T_3_, T_4_, and blood biochemical parameters in broilers subjected to low ambient temperatures [46]. Likewise, earlier work by Guo and colleagues reported that supplementation of Gln can improve thyroid hormone levels, serum albumin, and globulin levels of 7-day-old chicks under cold stress conditions [49]. These prior findings complement ours and demonstrate the significant role of supplemental dietary Gln under stress circumstances. Gln is recognized as a crucial precursor for the synthesis of AA, amino sugars, proteins, and several other biologically essential compounds, and it has also been shown to serve as a principal metabolic fuel for rapidly proliferating cells [50,51]. Prior research suggests that dietary Gln may enhance serum albumin and globulin levels in stressed animals, likely due to the addition of exogenous Gln, which increases protein synthesis efficiency in the body [50]. This may support our results with regard to enhanced serum albumin and TP levels. Thus, in the context of our research methodology, including dietary supplementation of Gln with early-age cold conditioning, we propose that the chickens in our present study exhibited improved metabolic conditions.

Our findings demonstrated an improvement in the antioxidant status of cold-conditioned chickens supplemented with dietary Gln, which were subjected to cold stress later in their lives. Serum SOD levels in treatments supplemented with dietary Gln, irrespective of cold conditioning, were significantly higher than in other treatment groups. Though, the control diet at standard temperature exhibited reduced levels of TAC and CAT enzymes compared to all other treatment groups. Previous research indicated that dietary Gln could alleviate stress situations, perhaps by enhancing the expression of antioxidant genes, such as CAT, SOD, and glutathione peroxidase [52]. Furthermore, Gln (via glutamate), along with other Aas, has been identified as a precursor to glutathione, which exists inside the cell in both reduced and oxidized forms [53]. Thus, Gln supplementation may be used to sustain elevated glutathione levels and prevent oxidative stress-related damage. Our present results align with those of Lou and colleagues, who indicated that adding a certain level of Gln into the diet under cold stress might substantially enhance the activities of glutathione peroxidase and CAT in the blood of broilers [54]. Furthermore, a previous study by Liu, Yang, Yao, Hu, Liu, Lian, Lv, Xu, and Li [46] found that dietary supplementation of Gln increased the antioxidant state of broilers during cold stress by enhancing blood SOD and glutathione peroxidase activity, corresponding with our findings. In a low-temperature environment, broilers are required to consume considerable energy to maintain their body temperature, thereby diminishing the energy available for other physiological functions. This reduction impairs the broilers’ capacity to eliminate free radicals, resulting in an accumulation of excessive free radicals. Therefore, dietary supplementation of Gln under such stress conditions may defend the body from free radicals, preserve glutathione levels, stabilize the integrity of cell membranes and proteins, and facilitate the repair and functional recovery of damaged cells, consequently enhancing the body’s antioxidant capacity [55,56]. This demonstrates that supplemental dietary Gln enhanced antioxidant status in cold-conditioned chickens and mitigated the reduction in antioxidant capacity induced by cold stress.

In the same context, several heat shock proteins (HSPs) are synthesized in animal bodies as a defense mechanism against stress. Under normal physiological conditions, HSPs are sustained at low levels; however, upon exposure to abrupt environmental temperature fluctuations, a substantial amount of HSPs is synthesized to bolster resistance to stress-induced damage [57]. HSPs function as protective proteins by binding to and stabilizing cytoskeletal proteins when subjected to denaturation by reactive oxygen species (ROS) [58]. Heat shock protein 70 (HSP70) is the most widely studied member of the HSP family due to its potentially protective properties against diverse stressors [59]. Our study demonstrated that treatment with 0.5% Gln, irrespective of cold conditioning, and cold-conditioned treatment with 0.3% Gln significantly elevated blood HSP70 levels compared to other treatment groups. This finding reinforces the hypothesis that a higher level of supplemental dietary Gln (0.5%) offers protective benefits under cold stress conditions, whereas the 0.3% dietary inclusion may require additional strategies, such as cold conditioning, to attain the best protection.

In the current study, dietary supplementation of Gln in cold-conditioned chickens has shifted cytokine levels during cold stress conditions. Cytokines, a category of soluble polypeptides that have small molecular weights, are believed to be involved in several biological processes such as immunomodulation, cellular proliferation, and repair of tissues by acting as intercellular mediators that stabilize certain mRNAs and enhance their translation [60]. Earlier literature indicates that cytokine production by the organism serves as a mechanism to respond to various environmental stimuli and has been shown to have a function in moderating stress-related immunological disorders [61]. Addressing Gln supplementation for cold-conditioned broilers, it is valuable to emphasize the nonnutritive function of Gln in the animal body. Its nutritive role is primarily regarded as a means for sustaining nitrogen and energy balance; however, apart from that role, it can also serve as a regulator of immune response and cellular activity [53]. Gln is a potent immune stimulant, as shown by different in vitro and in vivo investigations [62,63]. The immunomodulatory effect of Gln has been shown through the study of its metabolism, which serves as a substrate for lymphocytes [64]. It is a recognized precursor in purine and pyrimidine synthesis, which is required promptly during lymphocyte activation; moreover, the dependency of lymphocytes on Gln for the production of the cell surface activation marker CD25 (the alpha subunit of the IL-2 receptor) has been shown [65].

In addition, the adoption of cold conditioning at an earlier age as a strategy to enhance tolerance to cold stress later in life generates cold acclimation in poultry and provides added protection via various mechanisms [60]. Cold adaptation can boost immunological function and increase resilience to cold stress in organisms and reduce the harm inflicted by detrimental low ambient temperatures. Of these, the alteration in immune function may serve as an indicator for assessing an animal’s adaptation to cold. Previous research by Yang and colleagues reported that following two weeks of cold adaptation training, the cell-mediated immune function and disease resistance in mice were enhanced [66]. In poultry, Manning and Wyatt [67] found that broiler chickens developed cold adaptation after 14 days of cold exposure at 10 to 12 °C, resulting in enhanced resistance to Aspergillus flavus to a certain degree. Moreover, Su et al. [68] indicated that a rapid temperature decline of 10 °C elevated the expression levels of anti-inflammatory cytokines, IL-6 and IL-4, while suppressing the release of the proinflammatory IFN-γ. Intriguingly, at present, we have seen an elevation in blood levels of both proinflammatory and anti-inflammatory cytokines, suggesting further investigation. Furthermore, in the present study, the cold-conditioned treatments supplemented with 0.5% Gln showed an elevation in serum troponin-T level. Troponin-T serves as an effective biochemical marker for early cardiac injury prior to the onset of clinical and pathological alterations, exhibiting elevated levels in ascitic birds compared to healthy counterparts under cold stress circumstances [69,70]. While our hypothesis predicted that Gln supplementation for cold-conditioned broilers would serve as a defense against the detrimental effects of cold stress, the observed increase in troponin-T levels may suggest a biological reaction that contributes to cardiac contractility dysfunction. This change could possibly be explained by prior literature suggesting that thyroid hormones, in conjunction with basal metabolism, initially increase the power stroke of myosin contraction and cardiac output in cardiomyocytes, thereby enhancing blood flow to regions with elevated metabolic activity [71]. Thyroid hormones exert both extranuclear and nuclear actions in cardiomyocytes. The extranuclear impact pertains to protein synthesis and the activation of membrane transporters and channels, while its nuclear effect, which is is mediated by thyroid receptors, governs the transcription of cardiac genes sensitive to these hormones [72]. The mRNA of these cardiac genes encodes several proteins that influence myocardial contractility, such as troponin, a protein produced upon myocardial damage [73]. Thus far, the raised levels of thyroid hormones in the present study may have contributed to the increased troponin levels; however, further investigation is still required to verify these alterations.

## 5. Conclusions

Based on the obtained findings, it could be concluded that 0.3% and 0.5% dietary Gln supplementation and/or early cold conditioning increased broiler performance under cold stress, as seen in improved growth, antioxidant status, and immunological responses, while exerting relatively modest impacts on hematological and biochemical indicators. The interaction between dietary Gln and cold conditioning notably enhanced multiple indicators of growth, antioxidant status, and immunological response, indicating that these interventions could serve as complementary strategies to enhance broiler resilience to cold stress.

## Figures and Tables

**Table 1:** Ingredients and composition (%) of the basal diet (as-fed basis).

Ingredients	Starter (0–21 d)	Grower-Finisher (22–35 d)
Yellow corn	54.0	58.93
Soybean meal (44% CP)	34.12	30.25
Corn gluten (60% CP)	6.1	4.90
Soy oil	1.0	1.18
Limestone	1.65	1.60
Monocalcium phosphate	1.65	1.65
Common salt	0.45	0.45
Premix *	0.30	0.30
DL-Methionine, 98%	0.15	0.16
Lysine, Hcl, 78%	0.30	0.30
NaHCO_3_	0.28	0.28
Calculated composition		
DM%	89.70	89.70
ME, kcal/kg	2900.47	2950.85
CP%	23.02	21.00
EE%	3.48	3.78
CF%	3.64	3.47
Ca%	0.99	0.96
Total P%	0.75	0.73
Available P%	0.45	0.45
Lysine%	1.34	1.24
Methionine%	0.52	0.50
Threonine%	0.86	0.78

* Vitamin and mineral premix per kg of diet: vitamin A, 10000 IU; vitamin D3, 5000 IU; vitamin E, 80 mg; vitamin K, 3 g; thiamine, 3 g; riboflavin, 9 g; pantothenic acid, 15 g; folic acid, 2 g; pyridoxine, 4 g; niacin, 60 g; cobalamin, 20 mg; biotin, 0.15 mg; Fe, 40 mg; Cu, 15 mg; Mn, 100 mg; Zn, 100 mg; I, 1 g; Se, 0.35 g. DM: Dry matter; ME: Metabolizable energy; CP: Crude protein; EE: Ether extract; CF: Crude fiber; Ca: Calcium; Total P: Total phosphorus; Available P: Available phosphorus. The calculated glutamine level (%) in the basal diet was 2.15 in the Starter phase (0–21 days) and 1.96 in the Grower-Finisher phase (22–35 days).

**Table 2:** Effect of dietary glutamine level and early cold conditioning on growth performance of broilers.

Diet	Temp													
			Starter (0–21 d)				Grower-Finisher (22–35 d)				Overall performance (0–35 d)			
		Initial BW	BW, g	BWG, g	FI, g	FCR	BW, g	BWG, g	FI, g	FCR	BW, g	BWG, g	FI, g	FCR
Control	STD	44.40	484.4 ^d^	440.0	500.8	1.13	1595.6 ^d^	1111.2 ^c^	2382.6	2.14 ^a^	1595.6 ^d^	1551.2 ^d^	2883.4	1.86 ^a^
	Cold	44.60	514.8 ^c^	470.2	528.6	1.12	1778.8 ^c^	1264.0 ^b^	2402.2	1.90 ^b^	1778.8 ^c^	1734.2 ^c^	2930.8	1.69 ^b^
0.3% Gln	STD	44.40	549.8 ^b^	505.4	546.0	1.08	1907.2 ^b^	1357.4 ^a^	2480.4	1.82 ^b^	1907.2 ^b^	1862.8 ^b^	3026.4	1.62 ^b^
	Cold	44.00	580.4 ^a^	536.4	548.8	1.02	1972.4 ^a, b^	1392.0 ^a^	2610.2	1.87 ^b^	1972.4 ^a, b^	1928.4 ^a b^	3159.0	1.63 ^b^
0.5% Gln	STD	44.40	562.4 ^b^	518.0	549.4	1.06	1935.4 ^b^	1373.0 ^a^	2591.2	1.88 ^b^	1935.4 ^b^	1891.0 ^b^	3140.6	1.66 ^b^
	Cold	44.40	596.8 ^a^	552.4	563.0	1.01	2010.2 ^a^	1413.4 ^a^	2626.4	1.85 ^b^	2010.2 ^a^	1965.8 ^a^	3189.4	1.62 ^b^
SEM		0.372	5.24	5.23	5.27	0.004	34.20	29.53	5.79	0.048	34.20	34.25	10.01	0.032
Main Effects														
Diet														
Control		44.50	499.60 ^b^	455.10 ^b^	514.70 ^b^	1.13 ^a^	1687.20 ^b^	1187.60 ^b^	2392.40 ^b^	2.02^a^	1687.20 ^b^	1642.70 ^b^	2907.10 ^b^	1.77^a^
0.3% Gln		44.20	565.10 ^a^	520.90 ^a^	547.40 ^a^	1.05 ^b^	1939.80 ^a^	1374.70 ^a^	2545.30 ^a^	1.85 ^b^	1939.80 ^a^	1895.60 ^a^	3092.70 ^a^	1.63 ^b^
0.5%		44.40	579.60 ^a^	535.20 ^a^	556.20 ^a^	1.04 ^b^	1972.80 ^a^	1393.20 ^a^	2608.80 ^a^	1.87 ^b^	1972.80 ^a^	1928.40 ^a^	3165.00 ^a^	1.64 ^b^
SEM		0.400	2.63	2.75	4.72	0.007	23.42	21.78	2.71	0.042	23.42	23.76	6.81	0.026
Temperature														
STD		44.40	532.20	487.80	532.06	1.09	1812.73	1280.53	2484.73	1.95	1812.73	1768.33	3016.80	1.71
Cold		44.33	564.00	519.66	546.80	1.05	1920.46	1356.46	2546.26	1.87	1920.46	1876.13	3093.06	1.65
SEM		0.272	9.33	9.34	6.59	0.010	42.18	33.28	26.37	0.041	42.18	42.18	31.48	0.030
*p*-values														
Diet		0.820	<0.001	<0.001	<0.001	<0.001	<0.001	<0.001	<0.001	0.001	<0.001	<0.001	<0.001	<0.001
Temperature		0.867	0.026	0.013	0.038	0.037	0.021	0.029	0.075	0.051	0.021	0.021	0.059	0.031
Diet × Temp		0.820	0.014	0.857	0.063	0.072	0.002	<0.001	0.134	<0.001	0.002	0.002	0.279	0.001

^a, b, c, d^ Means with different superscripts within the same row differ significantly (*p* < 0.05). Gln: glutamine; STD: standard temperature; BW: body weight; BWG: body weight gain; FI: feed intake; FCR: feed conversion ratio.

**Table 3:** Effect of dietary glutamine level and early cold conditioning on hematological and serum biochemical parameters of broilers.

Diet	Temp							
		Hb, g/dL	PCV %	TP, g/dL	Albumin, g/dL	Globulin, g/dL	T_3_, ng/mL	T_4_, ng/mL
Control	STD	9.46	36.40	3.77	2.14	1.63	4.09	22.67
	Cold	10.66	37.00	4.46	2.67	1.79	4.47	23.96
0.3% Gln	STD	10.96	37.40	4.97	3.11	1.85	4.53	25.29
	Cold	10.72	37.80	5.35	3.49	1.85	5.06	26.62
0.5% Gln	STD	11.04	37.60	5.24	3.42	1.82	4.71	26.06
	Cold	9.46	38.00	5.43	3.53	1.89	5.21	27.06
SEM		0.137	0.395	0.178	0.146	0.082	0.107	0.346
Main Effects								
Diet								
Control		9.79 ^b^	36.70	4.11 ^b^	2.40 ^b^	1.71	4.28 ^b^	23.31 ^b^
0.3% Gln		10.81 ^a^	37.60	5.16 ^a^	3.30 ^a^	1.85	4.79 ^a^	25.95 ^a^
0.5%		10.88 ^a^	37.80	4.87 ^a^	3.48 ^a^	1.85	4.96 ^a^	26.56 ^a^
SEM		0.067	0.509	0.247	0.195	0.141	0.090	0.329
Temperature								
STD		10.28	4.44	24.67	4.66	2.89	5.76	4.41
Cold		10.70	4.91	25.87	5.08	3.23	6.99	5.09
SEM		0.171	0.094	0.477	0.210	0.176	0.186	0.176
*p*-values								
Diet		<0.001	0.142	<0.001	<0.001	0.619	<0.001	<0.001
Temperature		0.061	0.003	0.079	0.125	0.143	0.007	0.008
Diet × Temp		0.375	0.981	0.504	0.513	0.908	0.817	0.943

^a, b^ Means with different superscripts within the same row differ significantly (*p* < 0.05). Gln: glutamine; STD: standard temperature; Hb: hemoglobin; PCV: packed cell volume; TP: total protein; T_3_: triiodothyronine; T_4_: thyroxin.

**Table 4:** Effect of dietary glutamine level and early cold conditioning on the antioxidant status of broilers.

Diet	Temp				
		TAC, U/mL	CAT, U/mL	SOD, U/mL	HSP70, ng/mg
Control	STD	9.29 ^b^	3.71 ^b^	142.53	3.8 ^b^
	Cold	11.45 ^a^	5.60 ^a^	155.10	4.07 ^b^
0.3% Gln	STD	11.74 ^a^	5.73 ^a^	157.31	4.22 ^b^
	Cold	12.21 ^a^	6.20a	158.67	5.56 ^a^
0.5% Gln	STD	11.97 ^a^	6.07 ^a^	158.18	5.13 ^a^
	Cold	12.32 ^a^	6.32 ^a^	159.78	5.65 ^a^
SEM		0.398	0.391	2.73	0.116
Main Effects					
Diet					
Control		10.37 ^b^	4.65 ^b^	148.81 ^b^	3.97 ^b^
0.3% Gln		11.97 ^a^	5.96 ^a^	157.99 ^a^	4.89 ^a^
0.5%		12.15 ^a^	6.20 ^a^	158.98 ^a^	5.39 ^a^
SEM		0.107	0.369	0.193	0.216
Temperature					
STD		1.76	11.00	5.17	0.36
Cold		1.84	11.99	6.04	0.75
SEM		0.113	0.336	0.324	0.095
*p*-values					
Diet		<0.001	<0.001	0.009	<0.001
Temperature		0.586	0.014	0.022	0.019
Diet × Temp		<0.001	0.016	0.132	0.002

^a, b^ Means with different superscripts within the same row differ significantly (*p* < 0.05). Gln: glutamine; STD: standard temperature; HSP70: heat shock protein 70; TAC: total antioxidant capacity; CAT: catalase enzyme; SOD: superoxide dismutase enzyme.

**Table 5:** Effect of dietary glutamine level and early cold conditioning on the immune status of broilers.

Diet	Temp					
		IL2, pg/mL	IL4, pg/mL	IL10, pg/mL	IFN-γ, pg/mL	Troponin, ng/mL
Control	STD	3.77	23.82 ^d^	22.66 ^e^	4.88 ^e^	0.0848 ^c^
	Cold	4.52	25.19 ^d^	25.38 ^d^	5.13 ^e^	0.095 ^c^
0.3% Gln	STD	5.04	29.26 ^c^	26.52 ^c, d^	5.94 ^d^	0.235 ^c^
	Cold	6.73	39.04 ^a^	31.77 ^b^	7.37 ^b^	1.06 ^a, b^
0.5% Gln	STD	6.19	32.91 ^b^	28.51 ^c^	6.46 ^c^	0.768 ^b^
	Cold	7.17	40.55 ^a^	34.74 ^a^	8.46 ^a^	1.10 ^a^
SEM		0.152	0.305	0.619	0.059	0.002
Main Effects						
Diet						
Control		4.14 ^b^	24.50 ^b^	24.02 ^b^	5.01 ^b^	0.09 ^b^
0.3% Gln		5.88 ^a^	34.15 ^a^	29.15 ^a^	6.65 ^a^	0.64 ^a^
0.5%		6.68 ^a^	36.73 ^a^	31.63 ^a^	7.46 ^a^	0.93 ^a^
SEM		0.147	0.342	0.825	0.047	0.001
Temperature						
STD		152.67	37.13	5.00	28.66	25.90
Cold		157.85	37.60	6.14	34.92	30.63
SEM		2.52	0.376	0.295	1.11	0.704
*p*-values						
Diet		<0.001	<0.001	<0.001	<0.001	<0.001
Temperature		0.100	0.344	0.015	0.008	<0.001
Diet × Temp		0.111	<0.001	0.003	<0.001	<0.001

^a, b, c, d, e^ Means with different superscripts within the same row differ significantly (*p* < 0.05). Gln: glutamine; STD: standard temperature; IL2: interleukin 2; IL4: interleukin 4; IL10: interleukin 10; IFN-γ: interferon-γ.

## Data Availability

The original contributions presented in the study are included in the article; further inquiries can be directed to the corresponding author.
